# Characteristics of older adults admitted to the emergency department (ED) and their risk factors for ED readmission based on comprehensive geriatric assessment: a prospective cohort study

**DOI:** 10.1186/s12877-015-0055-7

**Published:** 2015-04-26

**Authors:** Mieke Deschodt, Els Devriendt, Marc Sabbe, Daniel Knockaert, Peter Deboutte, Steven Boonen, Johan Flamaing, Koen Milisen

**Affiliations:** Department of Public Health and Primary Care, Health Services and Nursing Research, KU Leuven, Kapucijnenvoer 35/4, 3000 Leuven, Belgium; Department of Geriatric Medicine, University Hospitals Leuven, Herestraat 49, 3000 Leuven, Belgium; Department of Public Health and Primary Care, Emergency Medicine, Kapucijnenvoer 35/4, 3000 Leuven, Belgium; Department of Emergency Medicine, University Hospitals Leuven, Herestraat 49, 3000 Leuven, Belgium; Department of Internal Medicine, University Hospitals Leuven, Herestraat 49, 3000 Leuven, Belgium

**Keywords:** Acute care, Comprehensive geriatric assessment, Emergency department, Readmission, Risk factors

## Abstract

**Background:**

Patients aged 75 years and older represent 12% of the overall emergency department (ED) population, and this proportion will increase over the next decades. Many of the discharged patients suffer an unplanned readmission in the immediate and midterm post-discharge period, suggesting under recognition of psychosocial, cognitive and medical problems. The aim of this study was to compare the characteristics of older patients admitted and discharged from the ED and to determine independent predictors for ED readmission 1 month and 3 months after ED discharge based on comprehensive geriatric assessment (CGA).

**Methods:**

Cohort study in a Belgian university hospital. A CGA, including demographic and medical data (e.g. reason for admission, comorbidity, number of medications), functional (e.g. activities of daily living, falls), mental (i.e. cognition, dementia, delirium), and nutritional status, and pain, was performed in 442 ED patients aged 75 years or older.

**Results:**

Patients discharged from the ED (n = 117, 26.5%) were significantly less dependent for ADL, mobility, shopping and finances compared with hospitalised patients. Hospitalised patients (n = 325, 73.5%) were significantly more at risk for having nutritional problems, had a higher comorbidity index, and a lower cognitive status compared with those discharged. Ninety-seven patients (82.9%) were discharged home from the ED. Of the latter, 18 (18.6%) and 28 patients (28.9%) suffered an ED readmission within 1 and 3 months, respectively. At one month post-discharge, nursing care at home, meals on wheels, and risk for depression; and at 3 months post-discharge previous hospitalisation in the last 3 months, physiotherapy and meals on wheels were found to be independent predictors for ED readmission, respectively.

**Conclusions:**

This study observed a geriatric risk profile in older adults at the ED and a high readmission rate of those discharged, and suggests the potential value of CGA in identifying older patients at high risk for ED readmission.

## Background

Patients aged 75 years and older constitute 12% of the overall emergency department (ED) population and this number is expected to increase dramatically over the next decades [1]. Compared to younger patients, older people have a higher accident severity rate, a longer ED length of stay, and a higher risk to be admitted to the hospital [2-5]. Furthermore, older patients at the ED have an increased risk for functional decline (10-45% after 3 months), institutionalisation (10% after 3 months), and mortality (10% after 3 months) [4].

As only 32–68% of geriatric patients who visit the ED are admitted to the hospital, a substantial proportion will be discharged after initial diagnosis and treatment at the ED [5]. However, many of these discharged patients suffer unplanned readmissions, suggesting under recognition of psychosocial, cognitive and medical problems during their initial ED visit, or insufficient follow-up or therapeutic resilience. ED readmission rates range from 12 to 20% at 1 month, from 19 to 24% at 3 months and are about 40% at 6 months post-discharge, respectively [1,6,7].

Older persons are characterized by an atypical presentation of symptoms and have complex medical and psychosocial problems that may complicate ED care [3,4,8]. Although functional decline and psychosocial problems are prevalent in geriatric patients, they are rarely recognized and documented during the ED admission [2,4]. Research demonstrated that physical limitations are ignored in 75% of the geriatric patients at the ED [4]. Cognitive deficits are present in 15 to 40% of older people admitted to the ED, but only half are recognized [4].

Because the biological model of acute in-hospital care has been proven inadequate to manage geriatric patients [6], innovative care models for older patients have been based on a holistic approach including comprehensive geriatric assessment (CGA). CGA has been defined as “a multidimensional interdisciplinary diagnostic process focused on determining a frail elderly person’s medical, psychosocial and functional capabilities in order to develop a coordinated and integrated plan for treatment and long-term follow-up” [9]. CGA based interventions have been proven to reduce mortality and institutionalisation in patients admitted to an acute geriatric ward [10]. At the ED, CGA has been found to identify two new geriatric problems on average per patient not detected by ED physicians [11], and has been found to decrease functional decline and ED readmissions [12]. Introducing a CGA based care model may therefore prove to be an effective strategy to avoid unnecessary and inappropriate ED readmissions in older patients. Hence, the aim of this study was to conduct a CGA in patients aged 75 years or older admitted to the ED to 1) compare the profile of hospitalised with non-hospitalised patients, and 2) determine independent predictors for ED readmission 1 month and 3 months post-discharge for non-hospitalised patients.

## Methods

### Study design

A single-center cohort study was conducted with follow-up at one and three months. After providing written and oral information, written informed consent or proxy consent (i.e. by the next of kin) was obtained for every participant before inclusion. The Medical Ethics Committee of the Leuven University Hospitals approved the study. (Identification number: B322201112405).

### Study setting and population

The study was conducted at the ED of the University Hospitals Leuven, Belgium. The ED consists out of an admission and treatment area (18 cubicles) and three observation care units (25 beds of which seven are equipped as intensive care beds). In 2012, 54280 patients visited the ED, of which 35% (n = 19.054) were admitted to the hospital. Persons aged 75 years or older constituted 16% (n = 8752) of all ED visits. Two out of three patients aged 75 years or older (66.3%, n = 5806) were referred to the ED by their general practitioner, and 66.0% (n = 5780) had to be admitted to the hospital.

**Figure 1:**
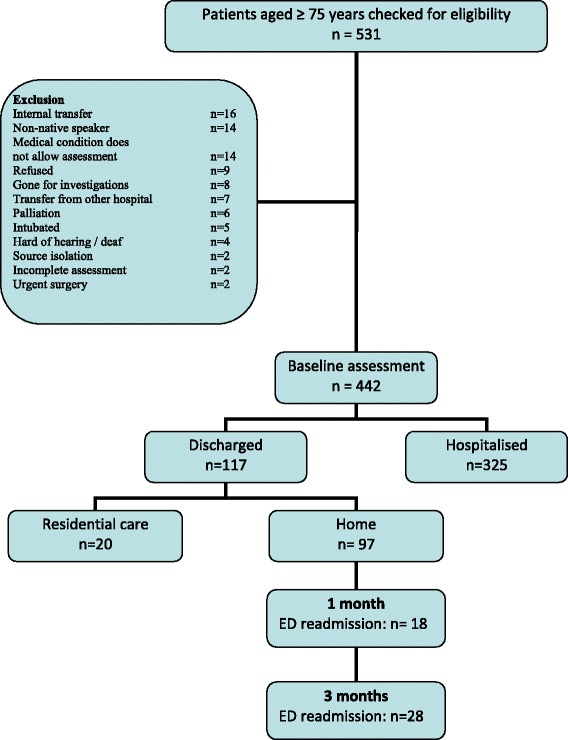
Study flowchart.

Dutch speaking patients aged 75 years or older were included in the study. Both community-dwelling people and nursing home residents were eligible for participation. Exclusion criteria are described in Figure 1. Eligible patients were asked to participate during a 5 hour time block on weekdays. In odd weeks patient recruitment was done between 8 am and 1 pm on Monday, Wednesday, and Friday, and between 1 and 6 pm on Tuesday and Thursday. In even weeks patient recruitment was done between 8 am and 1 pm on Tuesday and Thursday, and between 1 and 6 pm on Monday, Wednesday, and Friday. Patients were included from November 21st 2011 until February 10th 2012.

### Variables

#### Demographic variables

Data about age and gender was collected by reviewing the electronic patient file. Living situation (home alone, home together, nursing home) was determined by patient or proxy interview.

#### Preadmission variables

Functional status two weeks prior to admission was measured using the 6-item Katz Index of activities of daily living (ADL), retrospectively [13]. Dependence in bathing, dressing, transfer, toileting, incontinence, and feeding was assessed on a 3-point scale (1 = independent, 2 = partially dependent, 3 = fully dependent), resulting in a continuous total score ranging from 6 to 18. Instrumental activities of daily living, i.e. nursing care, home care, physiotherapy, meals on wheels, cleaning help, shopping assistance, help for finances, and use of a personal alarm system, was determined by patient or proxy interview. The number of home medications was registered by reviewing the electronic patient file or by patient or proxy interview.

#### Admission variables

Treatment priority was assessed with the Emergency Severity Index (ESI), a valid and reliable 5-level triage tool, ranging from ‘level 1’ immediate lifesaving intervention needed, ‘level 2’ patient needs to be seen as soon as possible, ‘level 3’ more than one resource needed, ‘level 4’ one resource needed, to ‘level 5’ no resources needed [14]. The ED triage nurse scored the ESI for patients admitted between 7.00 am and 10.00 pm.

The electronic patient file was reviewed by a nurse research assistant to determine patient’s comorbidity with the Modified Cumulative Illness Rating Scale (CIRS) [15]. The CIRS evaluates 14 biological systems as ‘0’ (i.e. the selected system corresponds to the absence of disorders), ‵1′ (i.e. slight (mild) abnormalities or previously suffered disorders), ‵2′ (i.e. illness requiring the prescription of medicinal therapy), ‵3′ (i.e. disease, which caused disability), and ‵4′ (i.e. acute organ insufficiency requiring emergency therapy). The total CIRS ranges from 0 to 56, with a higher score indicating a higher level of comorbidity.

Mobility was assessed by a research assistant with the Get Up and Go test (dependent versus independent with or without walking aid) [16], and by determining if the patient fell in the last year.

Pain was assessed with the pain thermometer by patient interview, a modified vertical Verbal Descriptor Scale alongside a graphic thermometer [17]. Scores range from 0 ‘no pain’ to 6 ‘worst possible pain’. Pain was considered as a score of 2 or higher on the pain thermometer.

Nutritional status was assessed by reviewing the electronic patient file and by patient interview using the Mini Nutritional Assessment (MNA), comprising six questions and a total score ranging from 0 to 14 [18]. A total score of 7 or less indicates malnourishment, 8 to 11 indicates risk for malnutrition, and 12 or higher indicates a normal nutritional status.

Cognitive status was measured by patient interview using the Sweet 16 [19]. The Sweet 16 instrument is scored from 0 to 16 (with 16 representing the best score) and includes 8 orientation items, 3 registration items, 2 digit spans, and 3 recall items. Occurrence of delirium was measured using the Confusion Assessment Method (CAM) [20]. A patient was considered delirious when an abnormal rating (‘acute onset’ and ‘fluctuation’) and ‘inattention’ and (‘disorganised thinking’ or ‘altered level of consciousness’) was recorded [21]. Risk for depression was assessed using the 3-question screening by Arroll et al. [22,23]. A patient was considered having a risk for depression if at least two out of three questions were answered positive. Risk for dementia was measured using the 16-item Informant Questionnaire of Cognitive Decline in the Elderly (IQCODE) [24]. Each item is a statement that needs to be scored by a close relative on a 5-point scale with 1 meaning ‘a lot better’, 2 ‘a little bit better’, 3 ‘no change’, 4 ‘a little bit worse’, and 5 ‘a lot worse’, compared to 10 years ago, respectively. The total score ranges from 16 to 80.

The electronic patient file was checked and the patient was interviewed to check whether the patient had any wounds or had been admitted to the hospital in the three months prior to the ED visit.

#### Follow up variables

The patient’s length of stay (LOS) at the ED and observational care unit was measured in hours and minutes. One and three months after ED discharge the patient or a relative was contacted by telephone and the electronic patient file was checked to determine patient’s discharge destination (hospitalised versus discharged: home alone, home together, or nursing home), mortality, or unplanned ED readmissions at the study or any other hospital. An unplanned ED-readmission was defined as “a subsequent or repeat ED visit that follows the initial ED visit or hospitalisation and cannot be foreseen at the time of ED or hospital discharge” [25].

### Procedures

Four research assistants were trained by a geriatric expert nurse (MD) in performing comprehensive geriatric assessments during a theoretical two-hour session and practice follow-up sessions in the hospital.

### Data analysis

Descriptive and comparative analyses were performed for all included patients and for the a priori determined subgroups (i.e. ‘discharged versus hospitalised patients’, and ‘patients readmitted versus not readmitted at the ED within 1 and 3 months after ED discharge’). Patients from residential care settings were excluded from the latter subgroup, as relevant data for instrumental ADL (e.g. shopping, finances) was missing in this group. Continuous variables were expressed as means with standard deviations for normally distributed data and medians with quartiles if non-normally distributed data was observed. Categorical variables were expressed as number of cases and percentages. Dichotomous or nominal variables were compared using the chi-square test, ordinal or nonnormally distributed continuous variables were compared using the Mann–Whitney U test and normally distributed continuous variables were compared using the Student t-test. To determine independent predictors for unplanned ED readmission at 1 month and 3 months in patients discharged home, all variables with P ≤ .20 in the univariate analyses were included in a multivariate backwards logistic regression model. Cases with missing data on any of the included variables were automatically removed from the final multivariate regression model. Multicollinearity was interpreted using tolerance values and variance inflation factors (VIF) values. Tolerance values of <0.10 and VIF values of >5 indicate a serious collinearity problem and in that case the results of logistic regression models should be interpreted with caution [26].

All analyses were performed using SPSS version 20.0 (SPSS Inc., Chicago, IL) All tests were two-tailed, assuming a 5% significance level.

## Results

### Study population

During the study period 531 patients were reviewed for eligibility. Eighty-nine patients did not fulfill the inclusion criteria, resulting in a total sample of 442 patients (Figure 1). The included patients had a mean age of 83.6 (SD 4.8) years and 60.4% were female (Table 1). The majority (86.0%) lived at home (alone or together) before ED admission. More than half (53.4%) had fallen at least once in the last year. Fifteen percent (n = 68) had wounds. The overall median LOS at the ED, including the stay at the observational care unit was 12h17min (Q1 8h03min; Q3 23h55min). At 3 months post-discharge one patient could not be reached by phone, resulting in a study sample of 441 patients at that time point.

**Table 1:** **Characteristics of the study sample and comparison between discharged and hospitalised patients**

**Variable**	**All patients (n = 442)**	**Discharged (n = 117)**	**Hospitalised (n = 325)**	**P-value**
Age, mean (SD)	83.6 (4.8)	83.1 (5.0)	83.8 (4.7)	.207
Female, n (%)	264 (60.4)	77 (65.8)	190 (58.5)	.163
Living situation, n (%)				.233
- Home, alone	155 (35.1)	37 (31.6)	118 (36.3)	
- Home, together	225 (50.9)	60 (51.3)	165 (50.8)	
- Residential care	62 (14.0)	20 (17.1)	42 (12.9)	
Nursing care at home, n (%)	123 (32.6)	25 (26.0)	98 (34.9)	.111
Home care, n (%)	48 (12.7)	7 (0.07)	41 (14.6)	.060
Physiotherapy, n (%)	40 (10.6)	10 (10.2)	30 (10.7)	.896
Meals on wheels, n (%)	59 (15.6)	10 (10.2)	49 (17.4)	.089
Cleaning help, n (%)	172 (45.4)	45 (45.9)	127 (45.2)	.902
Shopping assistance, n (%)	241 (63.8)	53 (53.0)	188 (67.6)	*.009*
Help for finances, n (%)	199 (52.5)	42 (42.0)	157 (56.3)	*.014*
Personal alarm system, n (%)	54 (14.0)	11 (11.1)	43 (15.0)	.333
ADL, Me (Q1-Q3)	7 (6–10)	6 (6–8)	7 (6–10)	*.045*
Number of medications at home, mean (SD)	7.8 (4.1)	7.2 (3.8)	8.0 (4.1)	.058
ESI, n (%)*				.188
- 1	1 (0.3)	-	1 (0.4)	
- 2	153 (39.7)	41 (39.0)	112 (40.0)	
- 3	199 (51.7)	47 (44.8)	152 (54.3)	
- 4	31 (8.1)	17 (16.2)	14 (5.0)	
- 5	1 (0.3)	-	1 (0.4)	
CIRS, mean (SD)	13.9 (4.9)	12.5 (5.1)	14.5 (4.8)	*<.001*
Get up and go independent, n (%)	281 (63.6)	91 (77.8)	190 (58.5)	*<.001*
Falls in the last year, n (%)	234 (53.4)	63 (54.8)	171 (52.9)	.734
Pain^$^, n (%)	171 (39.3)	45 (39.1)	126 (39.4)	.963
Nutritional status				*<.001*
- Malnourished	100 (22.6)	20 (17.1)	80 (24.6)	
- Risk for malnutrition	229 (51.8)	52 (44.4)	177 (54.5)	
- Normal nutritional status	113 (25.6)	45 (38.5)	68 (20.9)	
Sweet 16, mean (SD)	10.8 (3.8)	11.5 (3.8)	10.6 (3.8)	*.028*
Delirium, n (%)	35 (8.1)	5 (4.3)	30 (9.5)	.080
Risk for depression, n (%)	123 (29.4)	25 (22.7)	98 (31.8)	.072
IQ-code, mean (SD)	55.9 (9.5)	53.6 (8.9)	56.9 (9.6)	.055
Wounds, n (%)	68 (15.4)	17 (14.5)	51 (15.7)	.765
Last hospitalisation <3 months, n (%)	97 (22.9)	24 (20.9)	73 (23.7)	.538
Length of ED stay in hours, Me (Q1-Q3)	12 h17 (8 h03-23 h55)	8 h39 (5 h55-18 h32)	13 h41 (8 h49-25 h34)	*<.001*

$ Visual Analog Scale > 1; * 57 missings, ESI not given during nighttime; ADL = Activities of Daily Living; CIRS = Modified Cumulative Illness Rating Scale; MNA = Mini Nutritional Assessment; ESI = Emergency Severity Index; ED = emergency department.

### Discharged versus hospitalised patients

About one quarter (n = 117, 26.5%) of the patients were discharged, while three quarters (n = 325, 73.5%) were admitted to the hospital. Discharged patients were significantly less dependent for ADL, mobility (get up and go test), shopping and finances compared with hospitalised patients (Table 1). Hospitalised patients were significantly more at risk for having nutritional problems, had a higher comorbidity index and more cognitive problems (lower Sweet 16 score) compared with those discharged. The median LOS at the ED was significantly shorter in discharged patients (Me: 8 h39, Q1-Q3: 5 h55-18 h32) compared with patients that had to be hospitalised (Me: 13 h41; Q1-Q3: 8 h49-25 h34).

### Risk factors for ED readmission in patients discharged home

Of all patients discharged to their place of origin, 82.9% (n = 97) was discharged home, and 17.1% (n = 20) was discharged to a residential care facility. Of all 97 patients discharged home, 18 (18.6%) and 28 patients (29.2%) revisited the ED within 1 month and 3 months, respectively (Figure 1). In the univariate analyses (Table 2), readmitted patients differed significantly from non-readmitted patients: more of them were in need of nursing care at home, meals on wheels, cleaning help and shopping assistance, and more were at risk for depression. More than three times as many unplanned readmitted patients had already been hospitalised three month prior to the ED visit compared with non-readmitted patients. Also, patients readmitted within 1 month had a higher comorbidity index, while significantly more patients readmitted within 3 months fell in the last year (Table 2).

**Table 2:** **Risk factors for unplanned ED readmission in discharged patients based on univariate analysis**

	**1 month**			3 months*		
**Baseline variables**	**Readmitted (n = 18)**	**Not readmitted (n = 79)**	**P-value**	**Readmitted (n = 28)**	**Not readmitted (n = 68)**	**P-value**
Age, mean (SD)	84.1 (4.8)	82.2 (4.8)	.138	83.7 (4.3)	82.0 (5.0)	.124
Female, n (%)	11 (61.1)	52 (65.8)	.705	19 (67.9)	43 (63.2)	.667
Living situation, n (%)			.641			.715
- Home, alone	6 (33.3)	31 (39.2)		10 (35.7)	27 (39.7)	
- Home, together	12 (66.7)	48 (60.8)		18 (64.3)	41 (60.3)	
Nursing care at home, n (%)	10 (55.6)	15 (19.5)	*.002*	14 (50.0)	11 (16.7)	*.001*
Home care, n (%)	2 (11.1)	5 (6.4)	.489	4 (14.3)	3 (4.5)	.095
Physiotherapy, n (%)	3 (16.7)	7 (9.0)	.336	5 (18.9)	5 (7.5)	.132
Meals on wheels, n (%)	5 (27.8)	4 (5.1)	*.003*	6 (21.4)	3 (4.5)	*.010*
Cleaning help, n (%)	12 (66.7)	32 (41.0)	*.049*	18 (64.3)	26 (38.8)	*.023*
Shopping assistance, n (%)	14 (77.8)	37 (46.8)	*.018*	21 (75.0)	30 (44.1)	*.006*
Help for finances, n (%)	6 (33.3)	34 (43.0)	.450	12 (42.9)	28 (41.1)	.876
Personal alarm system, n (%)	1 (5.6)	10 (12.7)	.391	5 (17.9)	6 (8.8)	.207
ADL, Me (Q1-Q3)	7 (6–8)	6 (6–7)	.091	7 (6–8)	6 (6–7)	.100
Number of medications at home, mean (SD)	8.0 (3.7)	6.7 (3.8)	.195	7.9 (3.6)	6.7 (3.8)	.172
ESI, n (%)^			.868			.925
- 2	7 (41.2)	25 (36.2)		- 11 (42.3)	- 21 (35.6)	
- 3	6 (35.3)	35 (50.7)		- 10 (38.5)	- 31 (52.5)	
- 4	4 (23.5)	9 (13.0)		- 5 (19.2)	- 7 (11.9)	
CIRS, mean (SD)	13.8 (5.3)	11.1 (4.1)	*.021*	13.1 (5.5)	11.1 (3.8)	.091
Get up and Go independent, n (%)	14 (87.5)	70 (93.3)	.427	24 (92.3)	59 (92.2)	.985
Falls in the last year, n (%)	12 (66.7)	40 (50.6)	.218	20 (71.4)	32 (47.1)	*.029*
Pain^$^, n (%)	6 (33.3)	33 (41.8)	.510	9 (32.1)	30 (44.1)	.312
Nutritional status, n (%)			.903			.975
- Malnourished	2 (11.1)	9 (11.4)		3 (10.7)	8 (11.8)	
- Risk for malnutrition	7 (38.9)	35 (44.3)		12 (42.9)	30 (44.1)	
- Normal nutritional status	9 (50.0)	35 (44.3)		13 (46.4)	30 (44.1)	
Sweet 16, mean (SD)	13.1 (1.3)	12.4 (2.8)	.136	12.4 (2.4)	12.6 (2.7)	.730
Delirium, n (%)	0 (0)	1 (1.3)	.629	1 (3.6)	0 (0)	.120
Risk for depression, n (%)	8 (44.4)	12 (15.4)	*.006*	11 (39.3)	9 (13.4)	*.005*
IQ-code, mean (SD)	51.5 (2.6)	51.9 (6.8)	.894	52.9 (6.9)	51.5 (6.3)	.574
Wounds, n (%)	5 (27.8)	9 (11.4)	.074	6 (21.4)	8 (11.8)	.223
Last hospitalisation <3 months, n (%)	8 (44.4)	11 (13.9)	*.003*	11 (39.3)	8 (11.8)	*.002*
Length of ED stay in hours, Me (Q1-Q3)	7 h09 (5 h31-17 h28)	8 h34 (5 h47-16 h35)	.528	8 h24 (5 h54-22 h59)	8 h23 (5 h29-15 h32)	.256

Univariate analysis.

SD = Standard deviation; IQR = Interquartile range; ADL = Activities of Daily Living; CIRS = Modified Cumulative Illness Rating Scale; MNA = Mini Nutritional Assessment; ESI = Emergency Severity Index; ED = emergency department; * 1 patient loss-to-follow-up; $ Visual Analog Scale > 1, ^ ESI not scored during nighttime.

All variables with P < .20 in the univariate analyses (Table 2) were entered in the backwards logistic regression model (Table 3). Based on tolerance values and VIF values, no multicollinearity was found between these variables. A higher comorbidity index (OR 1.2; 95% CI 1.0 – 1.3), depression (OR 3.6; 95% CI 1.0 – 12.7), and hospitalisation in the 3 months prior to the ED visit (OR 4.5; 95% CI 1.3 – 16.2) were found to be independent predictors for ED readmission at 1 month before ED discharge. Meals on wheels (OR 6.6; 95% CI 1.8 – 15.5) and physiotherapy (OR 8.9; 95% CI 0.8 – 21.3) before admission, hospitalisation in the 3 months prior to the ED visit (OR 6.5; 95% CI 1.4 – 29.5), and falls in the last year (OR 3.0; 95% CI 1.0 – 9.2) were found to be independent predictors for ED readmission at 3 months follow-up (Table 3).

**Table 3:** **Risk factors for unplanned ED readmission in discharged patients based on multivariate backwards logistic regression**

**Follow-up**	**Variable**	**P value**	**Odds ratio (95% CI)**
1 month	Nursing care at home	0.023	4.1 (1.2 – 13.7)
	Meals on wheels	0.016	8.0 (1.5 – 43.1)
	Risk for depression	0.028	4.1 (1.2 – 14.3)
3 months	Last hospitalisation < 3 months	0.001	8.7 (2.4 – 32.4)
	Physiotherapy before admission	0.015	6.5 (1.4 – 29.5)
	Meals on wheels	0.023	8.6 (1.4 – 54.9)
	Fall in the last year	0.053	3.1 (1.0 – 9.4)

Multivariate backwards logistic regression.

## Discussion

ED readmissions are an economic marker for expensive care and may be considered a sentinel event questioning the quality of care during and after ED admission [27]. Because older patients with their multiple and complex needs have a high risk for early ED readmissions, the aim of this study was to compare the profile of older patients admitted and discharged from the ED based on CGA and to determine which CGA components are independent predictors for ED readmission at 1 month and 3 months follow-up.

Without studying the reasons for unplanned readmission, our study findings confirmed that readmission rates in older ED patients are high. At 3 months follow-up, the ED readmission rate (29.2%) was slightly higher compared to the 24% reported by McCusker et al. [28] and the 23.5% reported in a previous study conducted in the same hospital [7]. However, it was lower than the 38% reported in a Swiss study [29]. In the latter study, a historical cohort study was used and little was reported on selection criteria of the sample, which may explain the high incidence rate of readmissions.

Based on CGA, the results demonstrate that the profile of discharged patients, without studying the appropriateness of discharge, differs significantly from those admitted to the hospital. Older patients hospitalised through the ED are characterized by a higher dependency for (instrumental) ADL, malnutrition, cognitive deficits and a high comorbidity index, indicating a geriatric profile and confirming the hypothesis that the decision whether or not to admit a patient is not solely medical based. CGA, which include a social, cognitive, and functional evaluation, may support the decision making process regarding patient admissions. Although conducting a full or limited CGA as part of the initial assessment for older ED patients might seem a time-consuming process, it targets the at-risk population and may be a way to reduce readmission rate. Moreover with a median LOS for this older population at the ED (including the LOS on the observational care unit, often including buffer time before admission) of more than 12 hours, the argument that there is no time to conduct a CGA at the ED is waived. As mortality and mean hospital LOS correlate with increasing time spent at the ED, even when controlling for comorbid conditions [30], decreasing the LOS at the ED could be an objective for future intervention studies.

Nursing care, meals on wheels and physiotherapy at home, risk for depression, and hospitalisation in the 3 months prior to the ED admission were found to be independent predictors of ED readmission in patients discharged home, and a significant trend for falls in the last year was found. In addition, the descriptive analyses demonstrated that almost three out of four of the readmitted patients fell in the year before the initial ED visit. Therefore, assessment of social status and mental disorders, such as depression, and assessment of the functional status, including fall risk with appropriate referral to a falls and fractures clinic, seems of high importance. These elements should be taken into consideration when CGA interventions at the ED are designed and evaluated.

Research about CGA at the ED is scarce and considerable heterogeneity is observed between reported interventions. Descriptive research has demonstrated the feasibility of CGA interventions, and documented a large range of undetected care needs [31-34]. To the authors’ knowledge only three randomized controlled trials [35-37] and two pre-post implementation studies [29,38] reported outcomes of a geriatric intervention at the ED. Mion et al. reported a decrease in nursing home admissions after 30 days [35], while McCusker et al. found a reduction in four-month rate of functional decline. [36] Other significant outcomes in favour of the intervention group were detection of unknown medical problems, [38] more referrals to other healthcare disciplines [38], and a decrease in hospital admission and unplanned ED readmission rates [39]. On the other hand Basic et al. could not detect a significant reduction of admission to the hospital, length of inpatient stay, or functional decline during hospitalisation after an early geriatric assessment by geriatric expertise nurse in the ED [37]. Taking into account both experimental [35-37] and quasi-experimental [29,38-40] studies, the reasons for the lack of effectiveness on certain outcomes, seems to be, besides methodological considerations such as contamination and lack of power, failure to adhere to the recommendations made and target those who would most benefit from an intervention [37]. Positive studies on the other hand include geriatric expert nurses to perform the CGA under supervision of or in collaboration with a geriatrician [33-35], and the intervention has an important component of home-based follow-up [33-36]. When further testing the impact of CGA interventions at the ED, we need to take into account that CGA is preferably done by a multidisciplinary team including at least a geriatrician and a geriatric expert nurse. Further, the interventions should be performed in a high-risk population and should not be recommendation-based, and have a follow-up component at home, to have a higher impact [41]. The results of this study may contribute to the identification of that high-risk population, and improve the impact of CGA interventions at the ED.

The strengths of this study are its prospective design; the sample size; and limited loss-of-follow up, minimizing the risk for attrition bias. Some limitations should however be considered. First, not all patients could be included as the number of older adults presenting at the ED exceeded personnel resources. Although a strict recruitment protocol was followed, patients were not included at random and therefore selection bias has to be considered. Compared to the population of patients aged 75 years or older admitted at the study hospital in 2013, the included sample appeared to be slightly older and more ill as could be inferred from its higher treatment priority, longer LOS at the ED, and higher percentage of admitted patients. (Data available upon request). Also, we recruited in winter period, and did not recruit at night or during weekends. Therefore, we performed additional analyses on all patients aged 75 years or older admitted to the study hospital in 2013 (n = 8017) to check whether the patient profile for age, gender, treatment priority, time spent at the ED, and discharge destination differed significantly according to the time of the day (8 am – 8 pm versus 8 pm - 8 am), time of the week (Monday to Friday versus Saturday and Sunday), and time of the year (analysis per season). Patients admitted on weekdays had a slightly higher treatment priority (Pearson Chi^2^ = 11.2; P = .02) and spent more time at the ED (median 7 h30 versus 6 h57, respectively; P < .01), compared with those admitted in the weekend. During night time significantly more men were admitted at the ED (49.2% versus 45.9%, P = .03), the patients were slightly older (82.4 ± SD 5.1 versus 82.0 ± SD 5.1 year; p = .01), and spent more time at the ED (median 10 h47 versus 7 h11; P < .01). Treatment priority (P < .01) and median time spent at the ED was significantly different across the different seasons (summer 6 h37; spring 7 h36; autumn 7 h21; winter 7 h49; P < .01). Third, this was a monocenter study carried out at a university hospital. Patient characteristics in university and regional hospitals might differ. Fourth, the logistic regression model only refers to patients living at home. It would be interesting to conduct similar analyses for the nursing home population, however, the number of nursing home residents was too small. Despite these limitations, this study confirms the significant differences between discharged and admitted older patients, which could be the base to tailor diagnostic, therapeutic, and follow-up interventions aiming at preventing ED readmissions.

## Conclusion

This cohort study confirms the geriatric risk profile of older adults at the ED - especially in those hospitalised after the ED visit - and the high unplanned readmission rates at 1 and 3 months of those discharged from the ED. Nursing care, meals on wheels and physiotherapy at home, risk for depression, and hospitalisation in the 3 months prior to the ED admission, were found to be independent predictors for ED readmission at 1 or 3 months post-discharge, suggesting the importance of a proactive approach and integration of CGA based interventions at the ED to identify older patients at high risk for ED readmission.

## Abbreviations

ADL: Activities of daily living
CAM: Confusion assessment method
ED: Emergency department
ESI: Emergency severity index
CGA: Comprehensive geriatric assessment
CIRS: Cumulative illness rating scale
IQ-CODE: Informant questionnaire of cognitive decline in the elderly
LOS: Length of stay
MNAk: Mini nutritional assessment
VIF: Variance inflation factors
